# Supplementary material for: Comparative analysis of the microRNA transcriptome between yak and cattle provides insight into high-altitude adaptation
Source: PeerJ. 2017 Nov 2;5:e3959. doi: 10.7717/peerj.3959 (PMC5671665; doi:10.7717/peerj.3959)
Supplement: Figure S1 — (A) Comparable amount of miRNAs were identified using yak or bovine genome. (B–C) Hierarchical clustering and heat map matrix of pairwise Spearman’s correlations of the counts of bovine genome-annotated miRNAs (B) or yak genome-annotated miRNAs (C) between 11 miRNA libraries. [file peerj-05-3959-s001.pdf]

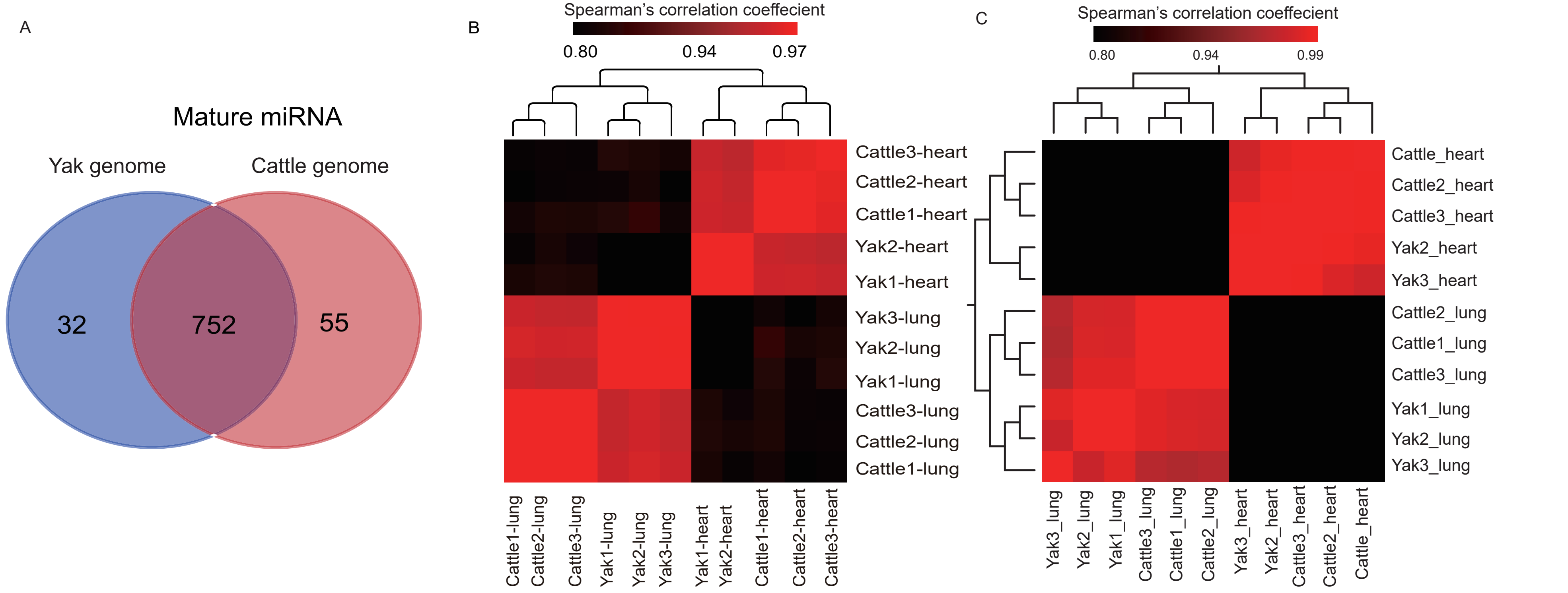
A
Mature miRNA
Yak genome
Cattle genome
32
752
55
B
Spearman's correlation coeffecient
0.80
0.94
0.97
Cattle3-heart
Cattle2-heart
Cattle1-heart
Yak2-heart
Yak1-heart
Yak3-lung
Yak2-lung
Yak1-lung
Cattle3-lung
Cattle2-lung
Cattle1-lung
Cattle1-lung
Cattle2-lung
Cattle3-lung
Yak1-lung
Yak2-lung
Yak3-lung
Yak1-heart
Yak2-heart
Cattle1-heart
Cattle2-heart
Cattle3-heart
C
Spearman's correlation coeffecient
0.80
0.94
0.99
Cattle_heart
Cattle2_heart
Cattle3_heart
Yak2_heart
Yak3_heart
Cattle2_lung
Cattle1_lung
Cattle3_lung
Yak1_lung
Yak2_lung
Yak3_lung
Yak3_lung
Yak2_lung
Yak1_lung
Cattle3_lung
Cattle1_lung
Cattle2_lung
Yak3_heart
Yak2_heart
Cattle3_heart
Cattle2_heart
Cattle_heart
