# Supplementary figures and images for: Comparative analysis of the microRNA transcriptome between yak and cattle provides insight into high-altitude adaptation
Source: PeerJ. 2017 Nov 2;5:e3959. doi: 10.7717/peerj.3959 (PMC5671665; doi:10.7717/peerj.3959)

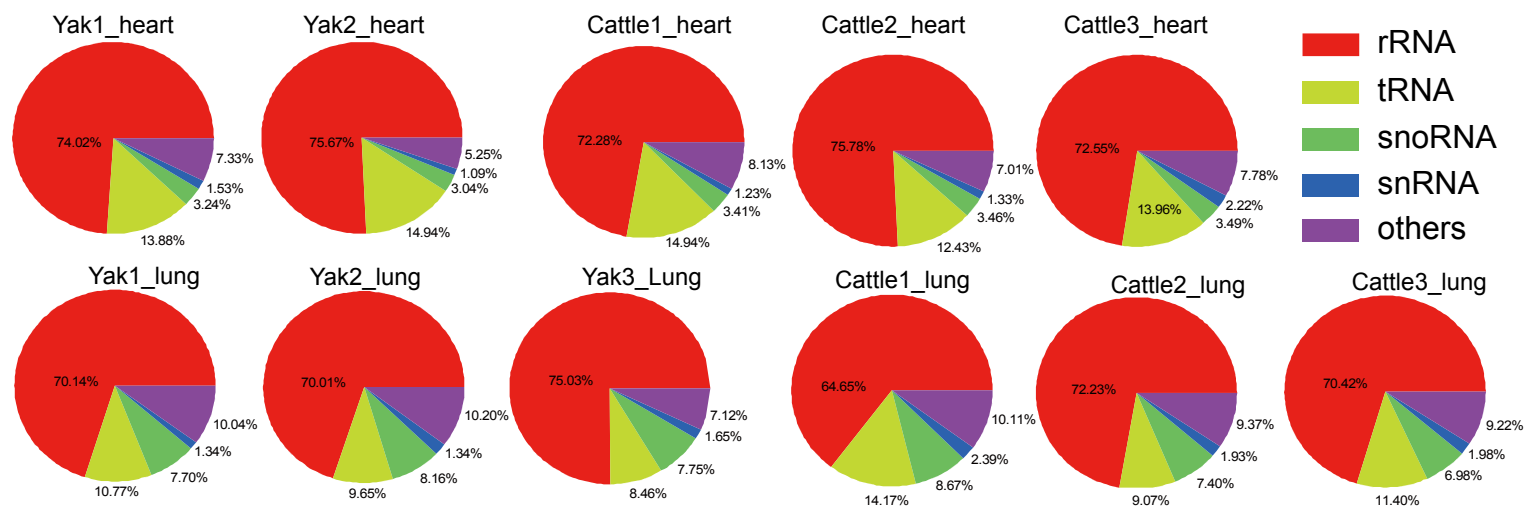

Supplement: Figure S2 [file peerj-05-3959-s002.pdf]

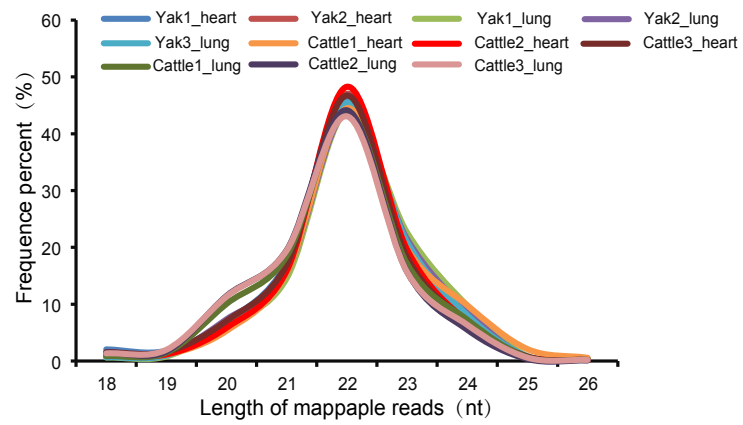

Supplement: Figure S3 [file peerj-05-3959-s003.pdf]

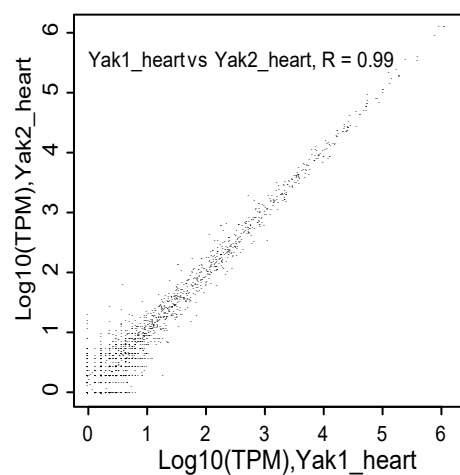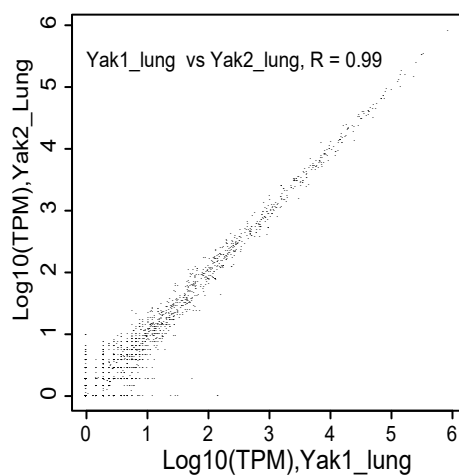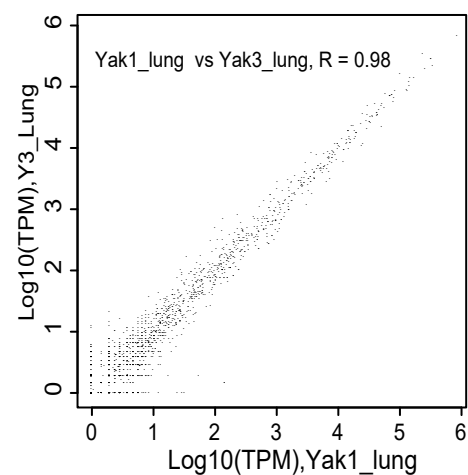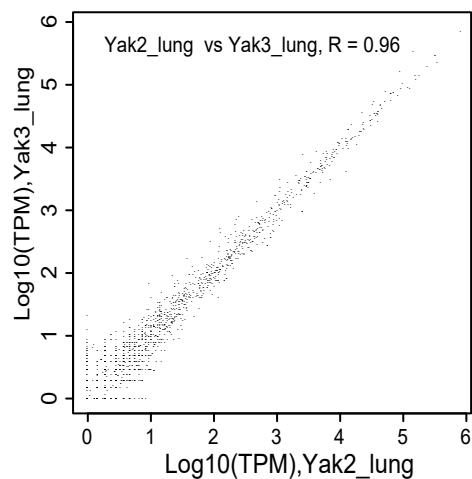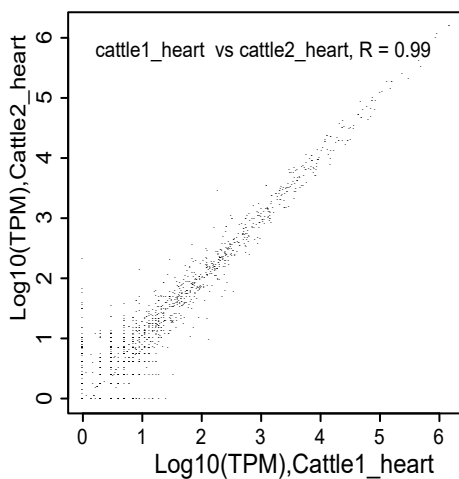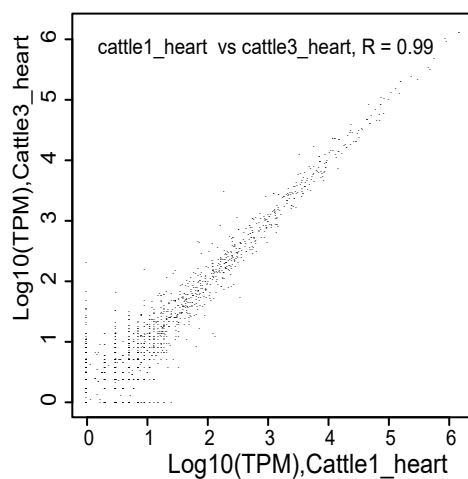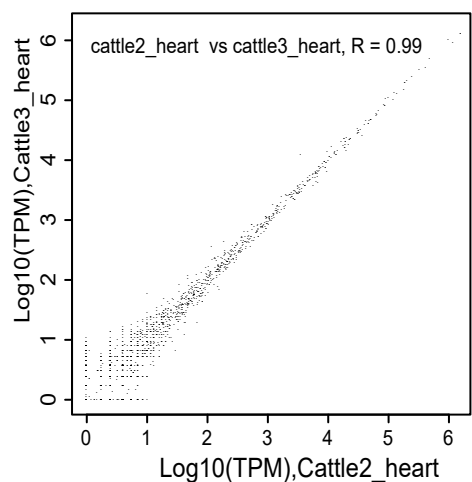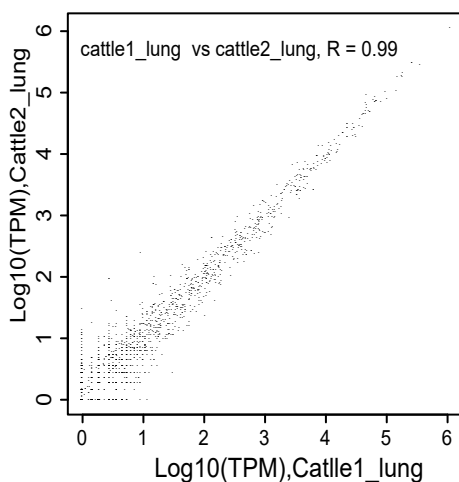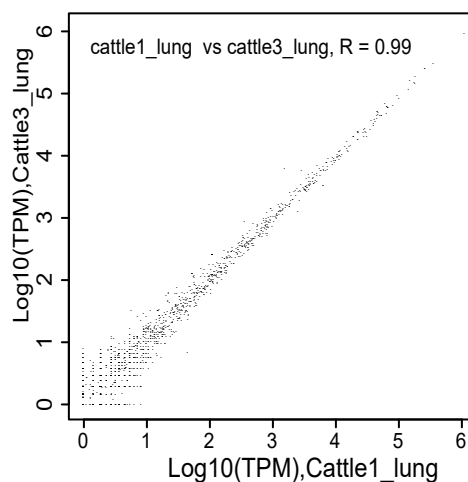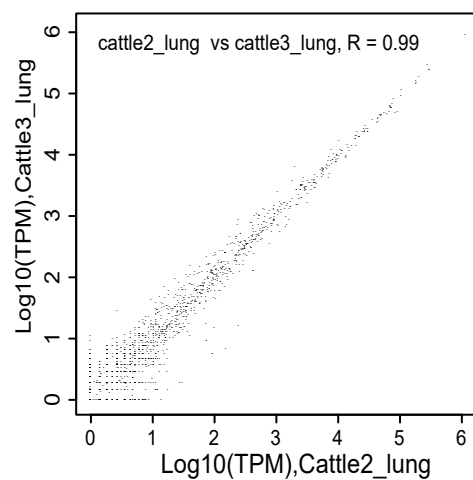

Supplement: Figure S4 [file peerj-05-3959-s004.pdf]

A

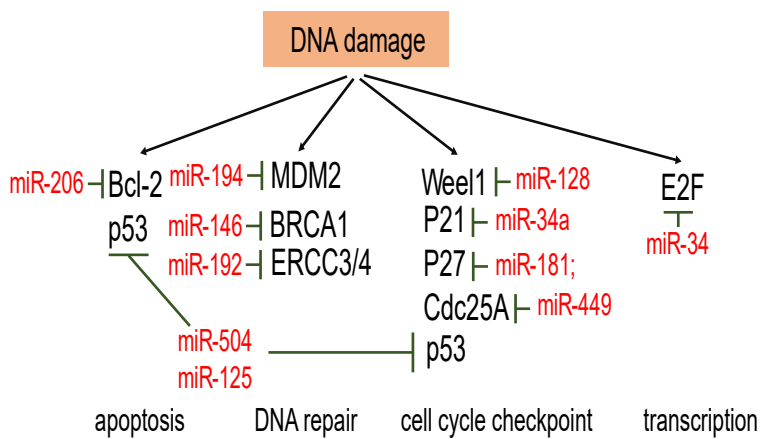

B

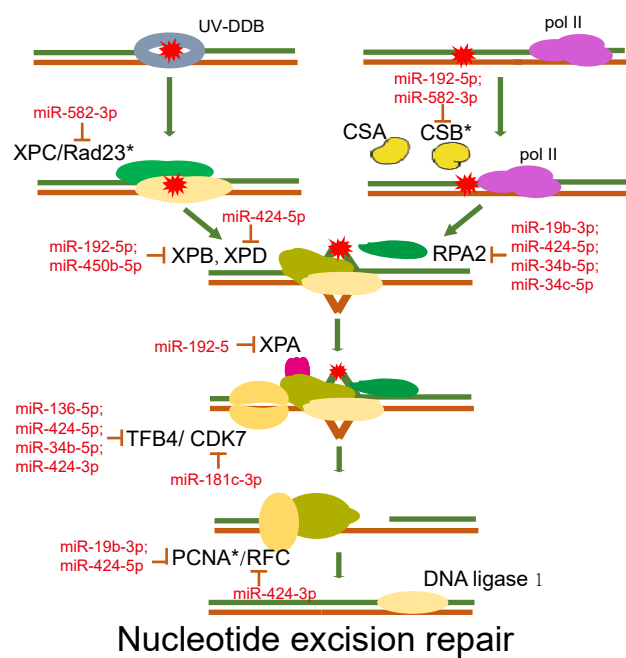

Supplement: Figure S5 [file peerj-05-3959-s005.pdf]
